# Viruses in the Heart: Direct and Indirect Routes to Myocarditis and Heart Failure

**DOI:** 10.3390/v13101924

**Published:** 2021-09-24

**Authors:** Colton R. Martens, Federica Accornero

**Affiliations:** Department of Physiology and Cell Biology, Dorothy M. Davis Heart and Lung Research Institute, The Ohio State University, Columbus, OH 43210, USA; colton.martens@osumc.edu

**Keywords:** cardiac inflammation, viral infection, myocarditis, cardiotropic viruses, heart failure

## Abstract

Viruses are an underappreciated cause of heart failure. Indeed, several types of viral infections carry cardiovascular risks. Understanding shared and unique mechanisms by which each virus compromises heart function is critical to inform on therapeutic interventions. This review describes how the key viruses known to lead to cardiac dysfunction operate. Both direct host-damaging mechanisms and indirect actions on the immune systems are discussed. As viral myocarditis is a key pathologic driver of heart failure in infected individuals, this review also highlights the role of cytokine storms and inflammation in virus-induced cardiomyopathy.

## 1. Introduction

Viral myocarditis is an increasingly appreciated cause of heart failure. Many viruses are known to infect the heart, and they can damage the heart both through direct viral processes (e.g., lysing of host cells or cleavage of host proteins by viral proteases) or through indirect mechanisms associated with the host immune response. While the immune response is necessary to fight the virus, it often causes severe cardiac damage and can sometimes be more damaging than the virus itself. The immune system mediates this damage through the release of harmful cytokines and through direct damage to uninfected cardiomyocytes by immune cells. The mechanisms involved differ depending on the individual and on the virus responsible. This review discusses the different viruses that have been implicated in myocarditis. It then covers the role of key pro-inflammatory cytokines and immune cells in mediating cardiac damage.

## 2. General Concepts Underlying the Cardiac Response to Viruses

Given that the immune system is both necessary to fight cardiotropic viruses and is a source of myocardial damage, the cardiac antiviral response must be carefully regulated. It must be rapid and efficient to minimize direct virus-mediated damage while not being excessive or prolonged so as to minimize indirect damage. The cardiac antiviral response typically progresses in multiple phases [1], although there is virus-specific variation in disease progression. In general, the initial acute phase of infection is characterized by viral replication and direct damage to the myocardium [2]. During this time, the virus is sensed by host factors called pattern recognition receptors (PRRs). These factors then stimulate the production of a wide array of antiviral proteins that serve to directly protect the cell and neighboring cells from infection and to recruit immune cells to fight the virus and facilitate tissue repair. In addition to the direct effect on the myocardium, early infection even of non-cardiac tissue can lead to a systemic increase in pro-inflammatory cytokines, and this so-called ‘cytokine storm’ may indirectly damage the heart [3]. Immune cells are first recruited to the myocardium during the early acute phase of infection. Innate immune cells arrive first, with neutrophils, natural killer (NK) cells, and macrophages arriving several days after infection in animal models [4,5]. These are followed by cells of the adaptive immune system that peak around one week after infection [6]. The second phase of myocarditis consists of both viral clearance and immune-mediated damage. Immune-mediated damage can result from the release of cardiomodulatory cytokines by immune cells, from the release of cytotoxic molecules that damage healthy cells, or from the presence of cardiac autoantibodies [7,8]. Depending on the specific viral species and the affected individual, the disease may either be resolved or progress into a chronic phase characterized by widespread fibrosis and persistent cardiac inflammation. While the majority of viral myocarditis patients recover normal cardiac function following infection, a significant fraction end up progressing to dilated cardiomyopathy and end-stage heart failure [9].

The multiphasic nature of viral myocarditis has made it difficult to develop treatments. During the early phases of infection where direct virus-mediated damage is the major source of myocardial damage, individuals may benefit from treatment with immunostimulants such as interferon beta (IFN-β) [9]. Conversely, during the chronic phase of infection where immune and inflammatory processes are the major source of myocardial damage, treatment with immunosuppressants may be more beneficial [9]. Treatment strategies also vary by virus, adding another layer of complexity to therapeutic development. Due to the fact that the efficacy of a given treatment depends on the specific virus involved and the stage in disease progression, the development of efficient therapeutics for viral myocarditis has been slow. The dominant treatment strategies remain supportive care and conventional heart failure treatment [9]. A more detailed understanding of the virus-specific variations in disease progression and of the broad factors that lead a subset of patients to progress to end-stage heart failure is necessary for the development of more efficient therapeutics. This review describes different viruses that are implicated in myocarditis as well as the role of cytokine storms and immune function in disease progression.

## 3. Cardio-Pathogenic Viruses

Many different viruses have been implicated in the development of myocarditis (Table 1). This includes enteroviruses, herpesviruses, parvoviruses, influenza viruses, and retroviruses. While there are virus-specific differences in the mechanisms by which they promote myocarditis, there are several general themes. As described above, most myocarditis-causing viruses promote cardiac damage by directly infecting cardiac cells and by promoting immune-mediated damage. Direct damage can result from infection of cardiomyocytes themselves or from infection of other cardiac cells such as endothelial cells or fibroblasts. Immune-mediated damage can result from excessive cytokine production or from the development of autoimmune myocarditis. Additionally, several viruses may promote myocarditis indirectly by suppressing the immune system of the host and thus increasing the chance that they are co-infected with additional, more pathogenic viruses. The different myocarditis-causing viruses that are discussed are listed in Table 1 along with their mechanisms of damage.

### 3.1. Enteroviruses

Enteroviruses are thought to be among the most common causes of viral myocarditis. Enteroviruses that have been implicated in viral myocarditis include echoviruses, poliovirus, and Coxsackie B viruses (CVBs). Of these, CVBs are the most studied. While the prevalence of cardiac infection by specific viruses has been difficult to estimate, CVBs are thought to be associated with 20–25% of myocarditis cases, making them one of the dominant etiological agents [30]. CVB infections are found worldwide, and they primarily occur in children and young adults. The course of myocarditis differs between individuals, with outcomes including complete recovery and disease resolution, cardiogenic shock, atrioventricular fibrillations and sudden cardiac arrest, the development of autoimmune myocarditis, and the onset of dilated cardiomyopathy [6,31,32]. The reason for such variation in outcomes is still not fully understood. 

The molecular details of CVB-associated myocarditis are well-known. Coxsackieviruses enter cardiomyocytes by binding to the co-receptors DAF (decay-accelerating factor) and CAR (coxsackievirus and adenovirus receptor) on the cell membrane [33]. Following internalization, the positive-sense single-stranded RNA genome is translated to produce a single polyprotein. The polyprotein is then cleaved by viral proteases to generate proteins necessary for replication, capsid formation, and cell lysis. Importantly, the viral proteases also cleave host factors to enhance viral proliferation and inactivate immune responses [33]. For example, CVB proteases cleave host translation initiation factors to promote cap-independent translation to favor viral translation [34,35]. CVB proteases can also cleave the key antiviral signaling protein MAVS (mitochondrial antiviral signaling protein), which abrogates the production of type 1 interferons (IFNs) [36]. CVB proteins also disrupt calcium signaling, endoplasmic reticulum function, and protein secretion, which results in severe cellular dysfunction [10,11]. Disruption of these processes may represent a strategy by the virus to enhance virion release and to prevent host myocytes from secreting cytokines to recruit immune cells [10]. Importantly, calcium signaling and endoplasmic reticulum function are important for cardiomyocyte contraction and intercellular signaling. These are essential for normal cardiac function, and the disruption of these processes by CVB constitutes another mechanism of direct, virus-mediated cardiac damage. 

The host response to CVB begins with the recognition of CVB RNA by several distinct host PRRs. These include toll-like receptor (TLR) 3, TLR7, and TLR8 [37,38]. These receptors then activate signal adaptor molecules such as TRIF (TIR domain-containing adaptor-inducing interferon-β) and MyD88 (myeloid differentiation primary response gene 88) [39,40,41], which ultimately promote the transcription of pro-inflammatory cytokines. Early cytokines include interleukin 1α (IL-1α), IL-1β, IL-6, tumor necrosis factor-α (TNF-α), and IFNγ [42]. Among other functions, these cytokines recruit innate immune cells such as NK cells and macrophages to the infected myocardium. This is followed by the recruitment of T cells to elicit a more specific antiviral response. While the antiviral response is necessary to limit direct virus-induced damage, immune cells can also cause indirect cardiac damage by inadvertently targeting healthy cells [7]. Additionally, CVB infection may promote autoimmune-mediated damage of the heart, possibly by driving immune reactivity toward cardiac myosin [12]. This combination of direct and immune-mediated damage can drive heart failure.

### 3.2. Herpesviruses

Another family of viruses commonly associated with viral myocarditis is herpesviruses. Herpesviruses are enveloped DNA viruses that typically cause latent infections in host cells. Cellular stress can activate these dormant viruses and result in virus-mediated damage. Herpesviruses implicated in viral myocarditis include human herpesvirus 6 (HHV-6), Epstein–Barr virus (EBV), cytomegalovirus (CMV), and varicella-zoster virus (VZV). HHV-6 has been detected in 16.5% of endomyocardial biopsy samples from patients with unexplained symptoms of heart failure [43]. Moreover, numerous cases of fatal myocarditis resulting from HHV-6 infection have been reported, supporting a causal role for HHV-6 in myocarditis [44,45,46]. Mechanistically, HHV-6 is known to infect cardiac endothelial cells and drive the production of pro-inflammatory cytokines [13]. This may contribute to the recruitment of immune cells to the myocardium and the development of cardiac inflammation during infection. Another possible mechanism by which HHV-6 could drive heart failure is by facilitating infection by additional cardiotropic viruses [45]. This is supported by the observations that HHV-6 can cause immunosuppression and that patients with myocardial HHV-6 are often co-infected with other cardiac viruses [14,15,43].

EBV has also been reported to cause viral myocarditis. Like HHV-6, EBV establishes latent infections in a large percentage of the population and infrequently causes disease. While rare, EBV can promote severe myocarditis [47,48,49,50]. While the mechanisms by which EBV promotes myocarditis remain poorly understood, EBV has been detected within cardiomyocytes of patients with inflammatory cardiomyopathy [16], suggesting that direct virus-mediated damage could contribute to disease development. Additionally, extreme lymphocytic infiltration of the myocardium has been shown in cases of fatal EBV infection [17], suggesting that indirect damage resulting from immune activity also plays a major role. Future research is required to determine the mechanisms of EBV-associated myocarditis.

CMV is another herpesvirus known to cause viral myocarditis. The exact prevalence of CMV-associated myocarditis is unknown, but CMV has been detected in up to 38% of post-mortem cardiac samples of patients with fatal myocarditis [51]. Like other cardiotropic viruses, CMV is thought to cause both direct and indirect cardiac damage. Insights into the mechanisms of CMV-driven cardiomyopathy come from mouse studies. Murine CMV (MCMV) is known to infect cardiomyocytes, fibroblasts, and cardiac endothelial cells in mice, and it has been shown to cause cardiomyocyte necrosis in vitro [18,52]. Additionally, mouse models have suggested a major role for the immune response in the development of CMV-associated myocarditis. Infection leads to rapid upregulation of pro-inflammatory cytokines such as TNF-α, IL-6, and INFγ [53]. Both CD4+ and CD8+ T cells are recruited within a week of infection [52,53], and T cells have been shown to be important for the development of myocarditis following MCMV infection in mice [19]. Autoantibodies targeting cardiac myosin are also thought to play a major role in CMV-induced myocarditis [20], as the transfer of MCMV-associated autoantibodies to uninfected mice is sufficient to cause myocarditis.

VZV is also known to cause myocarditis. VZV is the virus responsible for chickenpox, and infection is normally self-limiting. In rare cases, particularly in immunocompromised individuals and following heart transplants, VZV infection can cause myocarditis [54]. Cardiac infection by VZV can result in arrhythmias, pericardial effusion, and cardiac inflammation [21]. The mechanistic details of VZV infection remain poorly studied.

### 3.3. Parvoviruses

Parvoviruses are single-stranded DNA viruses, and within this family, parvovirus B19 is thought to be one of the most common viruses to cause myocarditis [55]. It infects the cardiac endothelium where it promotes apoptosis and upregulates a variety of pro-inflammatory cytokines [22,56,57]. Parvovirus B19 may also promote autoimmunity through the induction of autoantibodies [23], which may further contribute to myocardial damage following infection.

### 3.4. Retroviruses

Human immunodeficiency virus (HIV) infection is frequently associated with myocarditis, and this virus is the most predominant retrovirus associated with cardiomyopathies. HIV may promote myocarditis in part by causing direct cardiac damage, as the HIV genome has been detected diffusely in cardiomyocytes of HIV patients at autopsy [58]. Additionally, HIV has been shown to infect and promote the apoptosis of neonatal rat cardiomyocytes and coronary-artery-derived endothelial cells in vitro [24]. While these observations suggest that direct myocardial damage contributes to HIV-associated myocarditis, it is thought that indirect effects of HIV infection play a more significant role in disease progression. As with other cardiotropic viruses, HIV infection of the heart promotes the expression of pro-inflammatory cytokines and infiltration of the myocardium by macrophages and lymphocytes. Importantly, infiltrating immune cells are frequently infected with HIV with this virus having a high tropism for CD4+ T cells in particular, consistent with immunological disorders resulting from HIV infection [24]. Indeed, HIV may also promote viral myocarditis by causing immunosuppression and increasing one’s susceptibility to other cardiotropic viruses. This is supported by the observation that HIV myocarditis has been associated with cardiac co-infection with coxsackieviruses, herpesviruses, cytomegaloviruses, and parvoviruses [25]. HIV infection may increase infection by cardiotoxic bacterial species as well. Anti-retroviral medications used to treat HIV patients may also be cardiotoxic and thus result in an indirect association between HIV infection and myocarditis [59]. HIV infection is also thought to cause myocarditis indirectly by promoting atherosclerosis and subsequent cardiac stress, by causing nutrient deficiencies, and by causing autoimmune activation [60,61]. 

### 3.5. Influenza Viruses

Influenza viruses have long been associated with myocarditis. There are many case reports of influenza-associated myocarditis in humans [62,63,64,65,66,67], and myocarditis has been detected in up to 48% of fatal influenza cases [68]. Additionally, seasonal fluctuations in influenza infections have been observed to correspond to fluctuations in heart-failure-related hospitalizations, and influenza vaccination has been observed to reduce hospitalizations from cardiovascular disease [69]. These observations further support a role for influenza infection in cardiac pathology. Mouse studies have also shown the presence of influenza virus in the heart after infection, suggesting a possible direct role of influenza in the development of myocarditis [26]. Moreover, influenza A viruses have been shown to infect H9c2 cardiomyocytes in vitro and to drive the expression of pro-inflammatory cytokines such as IL-6, TNF-α, and IL-1β [27]. As will be discussed below, these cytokines contribute to myocarditis through a variety of mechanisms. Influenza viruses are also known to infect endothelial cells and cause vascular dysfunction, which may contribute to cardiac pathology indirectly. While these and other observations have firmly established influenza infection as a cause of viral myocarditis, the lack of an animal model for influenza myocarditis has made it difficult to understand the precise mechanisms through which influenza damages the heart. Certain genetically altered mouse lines have been shown to more accurately recapitulate myocarditis [26], and such models will play an important role in furthering our understanding of the details of influenza-associated myocarditis. 

### 3.6. Coronaviruses

Coronaviruses including the severe acute respiratory syndrome coronavirus 2 (SARS-CoV-2) may also cause myocarditis. SARS-CoV-2 infection is tightly associated with cardiomyopathy, as congestive heart failure is one of the most common comorbidities in infected individuals [70,71,72]. There have also been multiple reports of myocarditis developing in SARS-CoV-2 patients, and this is thought to be a major factor contributing to the mortality associated with coronavirus infection [71,72]. SARS-CoV-2 infection is also associated with an increase in circulating cardiac troponin, which is an indicator of cardiac damage [73]. Higher circulating cardiac troponin levels are associated with higher disease severity and mortality in COVID-19 patients [74]. COVID-19 patients also frequently develop electrocardiographic and echocardiographic abnormalities, and such abnormalities are associated with worse prognosis [75,76]. This further suggests that SARS-CoV-2 can promote myocarditis. Direct infection of cardiac tissue by SARS-CoV-2 is likely, as cells within the heart widely express the coronavirus receptor, angiotensin-converting enzyme 2. An early study has also reported possible SARS-CoV-2 in an endomyocardial biopsy sample from a patient with coronavirus-associated cardiogenic shock [77]. Additionally, SARS-CoV-2 has been shown to infect and drive apoptosis of human-induced pluripotent-stem-cell-derived cardiomyocytes in vitro [28]. Indirect effects of infection also probably contribute to SARS-CoV-2-mediated myocarditis. Systemic increases of pro-inflammatory cytokines such as TNF-α, IL-6, and C-reactive protein (CRP) are frequently reported in coronavirus patients [29]. These are critical factors involved in the pathogenesis of immune-mediated myocarditis, which suggests that SARS-CoV-2 also promotes indirect myocardial damage. 

## 4. Virus-Induced Cytokine Storm

It is clear that many distinct viruses can infect the heart and cause myocarditis. While many cardiotropic viruses cause direct myocardial damage, the development of myocarditis and subsequent cardiomyopathies is usually heavily influenced by the immune response. Viral infection results in the production of pro-inflammatory cytokines that can cause local and systemic inflammation that damages infected and uninfected tissues. Systemic inflammation associated with viral infection can lead to cardiac dysfunction even when the heart itself is not the primary site of infection. For example, pulmonary infection by SARS-CoV-2 can cause systemic increases in pro-inflammatory cytokines such as IL-6, CRP, IL-2, IL-7, TNF-α, MCP-1 (monocyte chemoattractant+ protein 1), and G-CSF (granulocyte-colony-stimulating factor), and the increase in pro-inflammatory factors correlates with cardiac injury during infection [3]. Such cytokines can adversely modulate cellular activity directly, drive apoptosis and trigger organ dysfunction, or recruit immune cells that can damage the tissue. The uncontrolled and excessive release of cytokines is called a cytokine storm, and it is a major source of mortality associated with infectious disease. 

The specific cytokines expressed during infection have not been clearly defined for each cardiotropic virus, and it is likely that there are virus-specific differences in the cytokine response elicited. Despite this complexity, there are some cytokines induced by a variety of cardiotropic viruses whose contributions to myocarditis are well-understood. This review highlights several of these cytokines, namely TNF-α, IL-6, and IL-1β.

### 4.1. Tumor Necrosis Factor Alpha

TNF-α is expressed by both immune and non-immune cells, and its systemic expression is increased following infection by a variety of cardiotropic viruses including influenza, CVB, and HIV. Cardiac TNF-α abundance during viral infection has been observed to correlate with reduced cardiac function in humans [78], and mouse models have demonstrated a causal role for TNF-α in virus-induced cardiac pathology. For example, mice that are resistant to CVB-mediated damage develop inflammatory lesions when treated with TNF-α [79]. Moreover, the addition of anti-TNF-α antibodies to infected mice reduces virus-induced cardiac pathology [80]. While these and other studies have shown that TNF-α contributes to the cardiac pathology that results from viral infection, TNF-α knockout (KO) mice have reduced viral clearance and higher mortality following infection. This likely reflects the importance of low-level or temporally and spatially isolated expression of TNF-α in mediating the response to infection, and the pathological effects of TNF-α may primarily result from its overproduction [81]. 

The molecular mechanisms of TNF-α-mediated cardiac dysfunction are relatively well-studied. TNF-α functions by binding to one of two receptors: TNFR1 or TNFR2. Activation of TNFR1 in cardiomyocytes promotes apoptotic signaling [82]. TNFR1 activation results in activation of c-Jun N-terminal kinase (JNK) pathways, which results in the activation of pro-apoptotic factors such as caspase-8, caspase-9, and caspase-3 [82]. TNF-α may also promote apoptosis by activating neutral sphingomyelinase, which is an enzyme that produces the lipids sphingosine and ceramide, both of which have been implicated in cardiomyocyte apoptosis [83]. TNF-α has also been observed to cause cardiomyocyte hypertrophy in mouse models with cardiac-specific TNF-α overexpression [84,85]. This is significant given that cardiac hypertrophy is a key step in the pathogenesis of heart failure. TNF-α is also known to drive extracellular matrix remodeling, which is another key process underlying myocarditis-induced cardiac dysfunction. More specifically, TNF-α reduces collagen expression by fibroblasts and dysregulates key factors necessary for extracellular matrix maintenance such as matrix metalloproteinases (MMPs) and tissue inhibitors of MMPs (TIMPs) [86]. This disruption of the extracellular matrix promotes left ventricular dilation and cardiac dysfunction. 

TNF-α also reduces cardiomyocyte contractility. Rats infected with TNF-α for 15 days developed reduced left ventricular function [87], and negative inotropic effects of TNF-α have been recapitulated in cell culture and ex vivo model systems [88]. Mechanistically, TNF-α appears to reduce contractility through multiple distinct mechanisms. The negative inotropic effect of TNF-α is in part attributable to the activation of neutral sphingomyelinase signaling, as sphingosine blocks the ryanodine receptor that is necessary for calcium release during contraction [89]. TNF-α also promotes the activation of nitric oxide synthase (NOS), which has a negative inotropic effect as evidenced by the fact that NOS inhibitors abrogate TNF-α-induced reductions in contractility [88]. The negative inotropic effects of TNF-α may also result in part from inhibition of β-adrenergic receptors. β-adrenergic signaling is the major pathway through which cardiac output is increased during stress, and TNF-α has been shown to blunt β-adrenergic responses in the heart [90]. Mechanistically, TNF-α increases the expression of G-protein-coupled receptor kinase 2 (GRK2), which contributes to the cellular desensitization to β-adrenergic signaling [90].

### 4.2. Interleukin-6

Another major cytokine induced by cardiotropic viruses is IL-6. IL-6 is a pleiotropic cytokine that can have pro-inflammatory or anti-inflammatory properties depending on the cell type and mode of signaling. It directly regulates the activity of a variety of immune cells, including macrophages, NK cells, T cells, and B cells [91]. IL-6 expression is higher in patients with heart failure, and IL-6 levels correlate with disease severity and poor prognosis [92]. IL-6 may adversely affect the heart through a variety of mechanisms. Mice overexpressing IL-6 and the IL-6 receptor have increased cardiomyocyte hypertrophy [82]. IL-6 also reduces cardiac contractility by increasing the expression of inducible NOS in cardiomyocytes [92]. IL-6 also modulates MMP activity, suggesting a role for IL-6 in extracellular matrix remodeling [82].

IL-6 also plays a key role in the cardiac antiviral response. IL-6 KO mice that are infected with CVB develop more severe chronic myocarditis [93]. Such mice do not have a problem clearing the virus but instead have heightened production of other pro-inflammatory cytokines and increased infiltration of immune cells [93]. This suggests widespread immune dysfunction in IL-6 KO mice during infection. Interestingly, overexpression of IL-6 also accelerates myocarditis in mice [94], suggesting that IL-6 levels must be carefully controlled to optimize the antiviral response. While the absence of IL-6 has been associated with increased chronic myocarditis during CVB infection, models of experimental autoimmune myocarditis have shown that IL-6 can promote autoimmune dysfunction. IL-6 KO mice that are immunized against a self-antigen (myosin heavy-chain peptide) are protected from autoimmune myocarditis as compared to wild-type mice [95]. IL-6 has also been implicated in a variety of other autoimmune diseases, including autoimmune encephalomyelitis and rheumatoid arthritis [96,97]. IL-6 may promote autoimmune disease by promoting the production of T-helper 17 (Th17) cells [98]. Th17 cells play an important role in the progression of autoimmune myocarditis, and it has been shown that the transfer of Th17 cells from mice with autoimmune myocarditis is sufficient to cause myocarditis in normal mice. IL-6 may also promote autoimmune disease by increasing the expression of cell adhesion molecules and increasing the recruitment of immune cells into tissues [98]. 

### 4.3. Interleukin-1 Beta

Another pro-inflammatory cytokine that is upregulated during viral infection is IL-1β. IL-1β is expressed primarily in monocytes and macrophages. It is initially produced as an inactive precursor, and it is activated and released following proteolytic cleavage by caspase-1 of the inflammasome complex [99]. IL-1β expression is increased in the hearts of mice with viral myocarditis, and its contribution to the pathogenesis of myocarditis is evident by the fact that addition of an IL-1 receptor antagonist reduces CVB-induced cardiac damage in mice [100]. Moreover, addition of exogenous IL-1β during CVB infection is sufficient to drive myocarditis in mouse strains that were previously resistant [79]. IL-1β adversely affects the myocardium through several mechanisms. As with TNF-α and IL-6, IL-1β has negative inotropic effects. Injection of mice with IL-1β results in a reduction in left ventricular fractional shortening [101]. Additionally, human serum from patients with septic shock reduces cardiomyocyte contractility, and immunodepletion of IL-1β from the serum attenuates this effect [102]. The mechanisms by which IL-1β reduces contractility include suppressing β-adrenergic signaling, blocking L-type calcium channels, driving the expression of NOS, and altering the activity of phospholamban and sarcoplasmic/endoplasmic reticulum calcium ATPase [99]. 

IL-1β has also been shown to drive cardiomyocyte apoptosis in vitro [103]. Treatment of cardiomyocytes with IL-1β results in increased expression of caspase-3 and increased release of cytochrome c from the mitochondria. Both of these processes support caspase-dependent apoptosis. IL-1β treatment was also shown to increase the expression of factors involved in caspase-independent apoptosis, such as endonuclease G. Expression of anti-apoptotic factors such as survivin and XIAP (X-linked inhibitor of apoptosis protein) is also downregulated in cardiomyocytes following IL-1β treatment [103]. Other mechanisms by which IL-1β adversely affects the heart include driving cardiomyocyte hypertrophy and extracellular matrix (ECM) remodeling [103]. The pro-hypertrophic effect of IL-1β has been shown in vitro, and it may result from an induction of the fetal gene expression program as is typically associated with hypertrophic growth [104]. IL-1β has been shown to alter extracellular matrix remodeling through multiple mechanisms. It reduces the proliferation of fibroblasts in vitro [105], and it has been shown to decrease collagen synthesis and increase the expression of multiple MMPs in cardiac fibroblasts [86].

**Figure 1:**
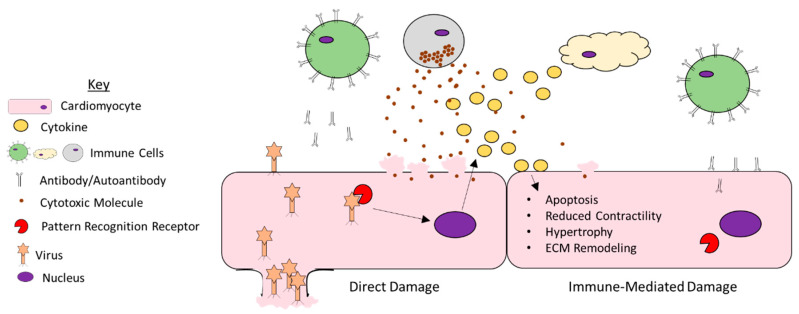
Mechanisms of viral myocarditis. A large number of viruses can infect cells of the heart and promote damage. They can promote direct damage by lysing host cells. Additionally, host cells are equipped with pattern recognition receptors that bind the virus and promote the expression of pro-inflammatory cytokines. While these cytokines can recruit immune cells that are necessary for viral clearance, they can also impair the function of uninfected myocytes by promoting apoptosis, reducing contractility, driving cardiomyocyte hypertrophy, and facilitating remodeling of the extracellular matrix. Furthermore, the immune cells recruited to the myocardium can damage uninfected myocytes through the release of cytotoxic molecules or through the production of autoantibodies.

Taken together, it is clear that diverse pro-inflammatory cytokines can directly disrupt cardiac function (Figure 1). Their heightened expression during viral infection is thus likely a major mechanism through which viruses damage the heart.

## 5. Role of Immune Cells in Viral Myocarditis

As discussed above, many cytokines involved in the pathogenesis of viral myocarditis adversely affect the myocardium by directly modulating the function of resident cardiac cells such as cardiomyocytes, endothelial cells, or fibroblasts. Pro-inflammatory cytokines also facilitate disease progression by recruiting immune cells and modulating their activity. Immune cells can damage the myocardium by targeting healthy cells for destruction or by releasing factors (e.g., more pro-inflammatory cytokines) that can negatively impact organ function. During cardiac infection, the early production of cytokines such as TNF-α, IFNγ, and IL-6 facilitates the recruitment of immune cells by upregulating the expression of cell adhesion molecules on nearby endothelial cells. Circulating leukocytes are then recruited to the site of injury to limit viral proliferation and initiate tissue repair. We now describe the key role of specific immune cells in the onset of viral myocarditis.

### 5.1. Neutrophils

Neutrophils are among the first immune cell types to reach the myocardium during infection [4,106]. Neutrophils defend against pathogens through a variety of mechanisms, including phagocytosis, the release of antimicrobial compounds, the release of reactive oxygen species (ROS), and the release of neutrophil extracellular traps [107]. Neutrophils also play an important role in the production of cytokines and the recruitment of additional immune cell types. The role of neutrophils in CVB-induced myocarditis has recently been investigated in a pair of studies [4,108] in which circulating neutrophils (LY6G positive cells) were immunodepleted in mice prior to infection. Xu and colleagues found that neutrophils were dispensable for the antiviral response and that survival, cardiac viral titers, and histopathological changes were all unaffected by neutrophil depletion [4]. In contrast, Rivadeneyra and colleagues found that neutrophil depletion actually lessened CVB-associated myocarditis [108]. Depletion of neutrophils resulted in reduced viral titers in the blood and heart, reduced histopathological signs of tissue damage, and reduced CVB-associated cardiomyocyte hypertrophy. The reason for the discrepancies between the two studies is unclear, although the use of different mouse lines (BALB/c in the former and C57BL/6 in the latter) could have contributed. Interestingly, it was also observed in the Rivadeneyra study that neutrophils themselves could be infected with CVB. While CVB was shown not to replicate inside infected neutrophils, CVB was shown to increase their expression of CD11b, which is necessary for the migration of neutrophils to destination tissues. Furthermore, CVB-treated neutrophils were found to have heightened ROS production, heightened activity of the antimicrobial compound myeloperoxidase, increased production of neutrophil extracellular traps, and increased expression of pro-inflammatory cytokines. It thus could be the case that CVB utilizes neutrophils to help distribute it throughout the body and that the beneficial effects of neutrophil depletion observed in this study are a consequence of the loss of this CVB-distributing mechanism and reduced intramyocardial presence of cytotoxic and pro-inflammatory neutrophils [108]. Additional investigation of the role of neutrophils in viral myocarditis is necessary.

### 5.2. Natural Killer Cells

Other early immune cells recruited to the myocardium during infection are NK cells and macrophages. NK cells have been shown to play an essential role in the cardiac antiviral response, as mice depleted of NK cells show higher viral titers in the heart and exacerbated myocarditis after CVB infection [109]. NK cells recognize infected cells and target them for destruction primarily by releasing perforin and other cytotoxic molecules. While this release is heavily regulated to minimize destruction of uninfected cells, perforin-mediated damage has been observed during viral myocarditis [7], and mice with a heterozygous perforin deletion experience reduced cardiac damage following viral infection [110]. Macrophages can be detected in the myocardium several days after infection in mouse models of viral myocarditis [111]. While the heart contains resident macrophages, most of the macrophage increase seen during acute inflammation is thought to result from recruitment of monocytes and subsequent differentiation into macrophages [112]. Macrophage depletion during CVB infection using liposome-encapsulated chlodronate led to increased viral titers in the serum and myocardium during the acute phase of infection, potentially indicating that macrophages play an important role in limiting viral proliferation [113]. Conversely, macrophage depletion also reduced cardiomyocyte necrosis and cardiac fibrosis, suggesting that macrophages positively contribute to chronic myocardial damage following infection. These observations suggest that the secondary myocardial damage caused by macrophages outweighs their apparent reduction in direct virus-mediated damage. The role of macrophages in the cardiac response to encephalomyocarditis virus has also been investigated by macrophage depletion, and it was observed that macrophage depletion led to reduced viral burden and dissemination [114]. This suggests that macrophages can be harmful in the context of this cardiotropic virus as well. 

### 5.3. Macrophages

There are different macrophage subpopulations that play distinct roles in cardiac inflammation. Macrophages are traditionally defined as M1 or M2 macrophages based on their mode of induction and functional characteristics. M1 macrophages (also called classically activated macrophages) can be induced by factors such as IFNγ and TNF-α and are characterized by the production of large amounts of pro-inflammatory cytokines. M2 macrophages (also called alternatively activated macrophages) can instead be induced by IL-10, IL-13, or IL-4 and primarily produce anti-inflammatory cytokines. These macrophage subpopulations have been shown to play important roles in mediating the heart’s response to viral infection. More specifically, the transfer of ex vivo generated M1 macrophages to CVB-infected mice has been observed to worsen myocarditis, while the transfer of ex vivo generated M2 macrophages has been shown to greatly ameliorate myocarditis [5]. Importantly, differences in macrophage polarization between male and female mice (with males biased toward M1 polarization and females biased toward M2 polarization) were observed, which may partially explain why males more frequently experience viral myocarditis. These studies reveal that macrophages play an important and multifaceted role in the pathogenesis of viral myocarditis.

### 5.4. B-Lymphocytes

The adaptive immune system, consisting of B and T cells, also plays an important role in viral myocarditis. B cells contribute to the immune response by producing antibodies, presenting antigens, and regulating the function of other immune cells. The role of B cells in the progression of viral myocarditis has been evaluated in several studies using mice that lack functional B cells (BcKO). Mena and colleagues [115] found that following CVB infection, BcKO mice were shown to have a delayed increase in viral titers in the heart as compared to immunocompetent mice, suggesting that B cells positively contribute to early viral replication. This was supported by the observation that a small percentage of B cells are infected with CVB in immunocompetent mice, which could indicate that B cells help disseminate the virus to target tissues. Importantly, despite the delayed increase in viral replication seen in BcKO mice, these mice were unable to clear the virus. They could, however, clear the virus when given B cells from CVB-immune mice, further supporting the importance of B cells in the antiviral response. Li and colleagues [116] also investigated the role of B cells in myocarditis using B-cell KO mouse strains. They found that BcKO mice had reduced histopathological severity of myocarditis following one week of CVB infection. It was also shown that loss of B cells led to a reduction in M2 polarized macrophages in the heart at seven days post-infection. These observations could be partially reversed in BcKO mice that received an adoptive transfer of B cells from CVB-infected wild-type mice one day before the BcKO mice were infected with CVB. While this study did not investigate viral clearance or chronic cardiac pathology, the results suggest that B cells may contribute at least to acute myocarditis by suppressing anti-inflammatory M2 macrophage populations. Importantly, some B-cell subpopulations are known to produce pro-inflammatory cytokines such as IFNγ and TNF-α that can encourage M1 polarization, while other B-cell subpopulations are known to produce anti-inflammatory cytokines such as IL-10 that can encourage M2 polarization [117]. It is thus likely that B cells play multiple distinct roles in the pathology of viral myocarditis and that unique B-cell subpopulations are involved in complex crosstalk with other immune cell populations. 

While B cells have been shown to be important for viral clearance, they have also been implicated in driving autoimmune myocarditis during infection. Many different cardiac autoantibodies have been reported in myocarditis models [118], and some have been suggested to contribute to disease development. For example, autoantibodies targeting cardiac myosin are observed in mice following infection with MCMV, and their transfer to uninfected mice results in cardiac inflammation and necrosis [8,20]. Despite their role in viral clearance and the apparent role of autoantibodies in disease progression, the precise roles of B cells and their subpopulations in viral myocarditis remain largely unknown. 

### 5.5. T-Lymphocytes

The contribution of T cells to viral myocarditis is well studied. T cells are divided into CD8+ T cells and CD4+ T cells. CD8+ T cells are comprised mainly of cytotoxic T cells, which recognize and destroy cells that present the proper antigen. They do so by releasing cytotoxic compounds such as granzymes and perforin, which facilitate apoptosis of the target cell [119]. CD4+ T cells are further differentiated into phenotypically distinct classes of helper T cells as well as regulatory T cells, which play diverse roles in the regulation of other immune cell populations. The roles of these different T-cell populations in the cardiac antiviral response have been dissected by many immune cell depletion studies. An early study using thymectomized mice and mice treated with anti-thymocyte antibodies found that T-cell depletion led to reduced necrosis in cardiac tissue and increased survival following CVB infection, suggesting a pathological role for T cells in viral myocarditis [120]. Similarly, infection of athymic mice with CVB results in less severe myocardial lesions as compared with normal mice [121]. The specific contribution of CD4+ and CD8+ T cells was investigated in a study using CD4 KO mice and β2-microglobin KO mice (β2mKO; β2-microglobin is a protein necessary for CD8+ T cell responses). β2mKO and CD4 KO mice both had increased survival and reduced cardiac viral titers following CVB infection compared with immunocompetent mice, again suggesting a pathological role for T cells during viral myocarditis. Despite the reduced mortality, CD4 KO mice (which are unable to mount normal helper-T-cell responses) had increased evidence of myocarditis at one week following CVB infection [122]. Depletion of CD8+ cells from these CD4 KO mice ameliorated the CD4 KO-induced increase in myocarditis, suggesting that CD4+ T cells may limit cardiac damage in part by suppressing the activity of cytotoxic T cells [122,123]. The mechanism through which cytotoxic T cells damage the myocardium is in part due to autoimmune targeting of healthy cardiomyocytes, as cytotoxic T cells from CVB-infected mice have been shown to kill uninfected cardiomyocytes in vitro and to cause severe myocarditis when adoptively transferred into T-cell-depleted CVB-infected mice [124]. 

Various CD4+ T cell populations contribute to myocarditis and to regulation of CD8+ T cell activity. Activation of T helper 1 (Th1) cells, for example, has been reported to drive myocarditis in part by activating CD8+ T cells [125]. Th1 cells are characterized by the production of IFNγ and other pro-inflammatory cytokines, and they have been associated with a variety of autoimmune diseases [126]. Th17 cells, which are also known drivers of autoimmune disease, have been implicated in the pathogenesis of viral myocarditis as well. The main function of Th17 cells is to produce IL-17, which activates neutrophils and stimulates the production of pro-inflammatory and anti-microbial factors in target cells [127]. Blocking the recruitment of Th17 cells to the myocardium through the administration of an anti-CCL20 antibody results in reduced severity of myocarditis following CVB infection [128]. Additionally, the ablation of a Th17-specific transcription factor (RORγt) that is necessary for Th17 differentiation confers resistance to experimental autoimmune myocarditis in mice [98]. The addition of anti-IL-17 antibodies following CVB infection has also been observed to greatly reduce myocarditis and cardiac viral titers [129,130]. Depletion of IL-17 during infection has also been shown to suppress the production of certain autoantibodies during infection [131], which suggests an involvement of Th17 cells in the pathogenesis of autoimmune myocarditis. Circulating Th17-cell and IL-17 levels are also increased in human patients with acute viral myocarditis, and the heightened IL-17 levels have been shown to correlate with B-cell activity [132]. This again suggests that Th17 cells positively contribute to B-cell-mediated autoimmunity during viral myocarditis. These pathological roles of Th17 cells in viral myocarditis make them a potential therapeutic target, and the addition of Th17-inhibiting factors such as IL-27, IL-35, IL-37, and progranulin has already been shown to reduce myocarditis in mouse models [133,134,135,136].

Regulatory T cells (Tregs) are another class of CD4+ T cells that regulate the cardiac antiviral response. Tregs are known for their role in self-tolerance and preventing autoimmune responses. Given that much of the damage done to the heart during viral infection is caused by immune hyperactivity and autoimmune activity, it is not surprising to find that Tregs limit the severity of myocarditis. Adoptive transfer experiments have demonstrated that Tregs reduced cardiac inflammation and viral titers one week after CVB infection [137]. Adoptive transfer of Tregs also reduces cardiac infiltration of pro-inflammatory monocytes, expression of proinflammatory cytokines, and cardiac functional deficits caused by CVB infection [138]. Immunodepletion and adoptive transfer experiments have also revealed that Tregs reduce cardiac fibrosis following CVB infection [138,139], which is important given the contribution of cardiac fibrosis to the electrical dysfunction and structural remodeling that accompany heart failure. Interestingly, the adoptive transfer of Tregs was found to reduce the expression of the Coxsackie co-receptor, CAR [137]. This suggests that Tregs may play a relatively direct role in limiting viral infection and dissemination in CVB-associated myocarditis. The mechanism of action of Tregs on CVB-associated myocarditis is likely multifaceted, as it has also been shown that secretion of the pleiotropic anti-inflammatory cytokine IL-10 also plays an important role in the amelioration of myocarditis by Tregs [139]. The beneficial effects of Tregs on viral myocarditis make them potential therapeutic targets, and factors that stimulate Tregs such as IL-37 and thrombospondin-2 have been shown to ameliorate myocarditis in animal models [135,140]. 

Taken together, it is clear that immune cells play an essential role in the progression of viral myocarditis. Many immune cell types have multifaceted roles in responding to cardiac infection, and whether they have pathological or ameliorative effects depends on the particular context (e.g., the viral species, the stage of infection, the local cytokine milieu). Nevertheless, it is clear that overactivation of certain immune cells increases the net damage done to the heart by cardiotropic viruses.

## 6. Treatment of Viral Myocarditis

The diversity of causative viruses has made it difficult to develop efficient treatments for viral myocarditis. Moreover, different treatment strategies may have different efficacies depending on the stage of disease in which they are applied. For example, immunosuppressant medications may be beneficial in the late stages of the disease where the bulk of cardiac damage comes from immune-mediated processes, but they may be harmful in the early stages when viral replication is the greatest source of damage. This has been exemplified by the anti-inflammatory medication prednisone, which has shown promise as a treatment for myocarditis in virus-negative patients [141]. Despite this benefit, mouse models have shown that the related compound prednisolone actually aggravates myocarditis if used in the early stages of infection [142]. Anti-inflammatory medications may also be considered for cases that progress to autoimmune myocarditis [143], which again highlights the fact that the utility of a given treatment is highly dependent on the nature of the specific case. 

In the case of myocarditis with active viral infection, antiviral medications or immunostimulants may be of benefit. For example, the antiviral medications artesunate, ganciclovir, and valganciclovir have been used to treat myocarditis caused by herpesviruses [43,144]. Additionally, IFN-β has been used as an immunostimulant to treat enterovirus-associated myocarditis in patients where viral genomes are present [145]. Again, while immunostimulants may benefit the individual during active infection, this is likely detrimental in contexts where the immune system is causing damage.

In light of the difficulties in developing treatments for viral myocarditis, it is important that steps are taken to limit initial infection. It is well known that a major predisposing factor to CVB-induced myocarditis is selenium deficiency, and other antioxidant micronutrients have been implicated as well [146]. More broadly, it appears that cardiac redox stress is a general phenomenon that can exacerbate disease induced by myocarditis-causing viruses [147]. Given that cardiac redox stress is sensitive to a large number of environmental parameters (e.g., diet, physical activity levels, pollution exposure, etc.), there are potentially many steps that one can take to reduce the risk of infection. Moreover, immune function, the integrity of barrier defense systems, and the conduciveness of individual cells to infection are all intimately connected with one’s lifestyle and environment [148], further suggesting that interventions aimed at lowering people’s risk of infection could be a successful prophylactic strategy to limit the harm caused by viral myocarditis. 

## 7. Conclusions

Many viruses can infect the heart and cause myocarditis. They can directly damage the heart by lysing infected host cells and disrupting cellular function. They can also cause indirect damage by promoting the expression of pro-inflammatory cytokines and the recruitment of immune cells. Several immune cell types are essential for clearing the virus and thus limit direct damage, but many immune cells have also been shown to exert pathogenic effects on the heart during infection. Autoreactive T cells and autoantibodies also play an important role in myocarditis and the progression to heart failure, and it is clear that the heart must carefully balance the immune response such that viral clearance is maximized while immune-mediated myocardial damage is minimized. The fact that damage can come from both the virus and the immune system has made it difficult to develop efficient treatments for viral myocarditis. Immunostimulants may increase the clearance of the virus and be beneficial in the early stages of infection at the cost of potentially exacerbating immune-mediated damage. Conversely, the use of immunosuppressants may attenuate immune-mediated damage while allowing increased viral proliferation and direct damage. A key step in the development of therapeutics will be understanding the virus-specific differences in the immune response and understanding the molecular mechanisms by which the host responds to infection. More attention should also be given to the extreme variation in outcomes between patients. The fact that some patients infected with a given virus remain asymptomatic or make a full recovery while others experience fatal myocarditis suggests that there are key host attributes that dictate differential outcomes. Identifying these factors could help us limit the harm caused by cardiotropic viruses in the future.

## Figures and Tables

**Table 1:** Viruses known to induce cardiomyopathies and their mechanisms of action.

Virus	Mechanisms of Cardiac Damage	References
Coxsackievirus B	Lysing cardiomyocytes; disrupting cardiomyocyte function; immune-mediated damage	[2,7,10,11,12]
Human Herpesvirus 6	Infection of cardiac endothelial cells; immune-mediated damage; immunosuppression	[13,14,15]
Epstein–Barr Virus	Direct cardiomyocyte infection; lymphocytic infiltration into the heart	[16,17]
Cytomegalovirus	Infection of cardiomyocytes, fibroblasts, and endothelial cells; T-cell-mediated damage; autoantibody stimulation	[18,19,20]
Varicella-zoster Virus	Cardiac electrical disruption; cardiac inflammation	[21]
Parvovirus B19	Infection of cardiac endothelial cells; autoimmune activation	[22,23]
Human Immunodeficiency Virus	Infection of cardiomyocytes; immune-mediated damage; immunosuppression and increased susceptibility to other cardiotropic viruses	[24,25]
Influenza Virus	Direct myocardial damage; immune-mediated damage; mostly unknown mechanisms	[26,27]
Severe Acute Respiratory Syndrome Coronavirus-2	Direct myocardial damage; overexpression of harmful cytokines	[28,29]

## Data Availability

Not applicable.
